# Supplementary material for: Molecular epidemiology of Animal African Trypanosomosis in southwest Burkina Faso
Source: PLoS Negl Trop Dis. 2022 Aug 22;16(8):e0010106. doi: 10.1371/journal.pntd.0010106 (PMC9436040; doi:10.1371/journal.pntd.0010106)
Supplement: Attachment — Submitted filename: Response to reviewers PLOS-NTD.pdf [file pntd.0010106.s001.pdf]

PLOS Neglected Tropical Diseases **<em@editorialmanager.com>**

CC: pbuscher@itg.be, boshart@lrz.uni-muenchen.de, boshart@lmu.de

Dear Dr Boulangé,

Dear Editor,

Thank you very much for submitting your manuscript "Molecular epidemiology of Animal African Trypanosomosis in southwest Burkina Faso" for consideration at PLOS Neglected Tropical Diseases. As with all papers reviewed by the journal, your manuscript was reviewed by members of the editorial board and by several independent reviewers. In light of the reviews (below this email), we would like to invite the resubmission of a significantly-revised version that takes into account the reviewers' comments.

As specified by Reviewer 2, all primary data have to be provided in an appropriate format (e.g. a source data file) for transparence and documentation.

All numerical data were submitted to a data depository at Cirad called DataVerse, and can be accessed following the link https://doi.org/10.18167/DVN1/OBRN6E Although we intended to submit also gels for PCR, and we requested extra time for that purpose, it soon became clear that it was unpractical. There are more than 500 gels, whose settings are manually recorded on a log book. Not only labelling each gel using a graphic program would have required not days but weeks, the same lengthy procedure would have had to be repeated when giving the description of each gel on the DataVerse. We provided instead as supplementary material a sample of gels for each pair of primers, hoping this would suffice for the reviewers and any interested party to get a visual idea of the results.

We cannot make any decision about publication until we have seen the revised manuscript and your response to the reviewers' comments. Your revised manuscript is also likely to be sent to reviewers for further evaluation.

This is well understood, we consider the reviewers’ requests totally legitimate, particularly concerning the absence of a suitable database.

When you are ready to resubmit, please upload the following:

[1] A letter containing a detailed list of your responses to the review comments and a description of the changes you have made in the manuscript. Please note while forming your response, if your article is accepted, you may have the opportunity to make the peer review history publicly available. The record will include editor decision letters (with reviews) and your responses to reviewer comments. If eligible, we will contact you to opt in or out.

Minor changes in the text were explained as comment in the track change version.

[2] Two versions of the revised manuscript: one with either highlights or tracked changes denoting where the text has been changed; the other a clean version (uploaded as the manuscript file).

Done

Important additional instructions are given below your reviewer comments.

Please prepare and submit your revised manuscript within 60 days. If you anticipate any delay, please let us know the expected resubmission date by replying to this email. Please note that revised manuscripts received after the 60-day due date may require evaluation and peer review similar to newly submitted manuscripts.

We had requested an extra month to suitably prepare the database and thoroughly double-check the data.

Thank you again for your submission. We hope that our editorial process has been constructive so far, and we welcome your feedback at any time. Please don't hesitate to contact us if you have any questions or comments.

Sincerely,

Yours faithfully,

Alain Boulangé, corresponding author

Philippe Büscher, PhD
Associate Editor
PLOS Neglected Tropical Diseases

Michael Boshart
Deputy Editor
PLOS Neglected Tropical Diseases

****

Reviewer's Responses to Questions

Key Review Criteria Required for Acceptance?</br></br>
As you describe the new analyses required for acceptance, please consider the following:</br></br>

Methods</br></br>
-Are the objectives of the study clearly articulated with a clear testable hypothesis stated?</br>
-Is the study design appropriate to address the stated objectives?</br>
-Is the population clearly described and appropriate for the hypothesis being

tested?</br>
-Is the sample size sufficient to ensure adequate power to address the hypothesis being tested?</br>
-Were correct statistical analysis used to support conclusions?</br>
-Are there concerns about ethical or regulatory requirements being met?</br></br>

Reviewer #1: The manuscript presents a comprehensive epidemiological study of three regions (Hauts-Bassins, Cascades and Sud-Ouest) located in the southwestern part of Burkina Faso, between April and May 2019, at the end of the dry season. The study included 1,898 domestic animals (cattle, sheep, goats and pigs). Various techniques were used to investigate the prevalence of infection of the trypanosomes responsible for AAT, transmitted by tsetse flies: T. congolense, T. vivax and T. brucei brucei. The presence and species of tsetse flies were also assessed.
The diagnostic methods used in the survey were adequate: PCR with TRYP1(R & S) primers to amplify the ITS1 of the ribosomal DNA of most trypanosome species, which allows species discrimination based on size polymorphism. Species-specific PCR tests were then used on the positive samples: T. brucei s.l. 171 (TBR1/TBR2), T. congolense "Savannah" type (TCS1/TCS2) (20), T. congolense "Forest" 172 type (TCF1/TCF2), T. vivax (TVW1/TVW2) (21) and T.b. gambiense specific glycoprotein 173 TgsGP ".
Serological diagnosis: whole soluble antigen of T. vivax, T. congolense or T.b. brucei and immune trypanolysis test.
The entomological survey used biconical traps for tsetse flies at various sites along the riverbanks in the three study areas for 48 hours each. This was followed by species identification. counting and determination of "the number of tsetse captured per trap per day".
Data analysis and statistical tests were performed to compare: 1. prevalence of the different study areas, the animal species, and the sexes 2. prevalence between the different infections and the association of trypanosome infections with anemia and 3. to study the effects of parameters such as sex, age, PCV, animal species, and study areas on the molecular prevalence. Finally, a Ven diagram was constructed to analyze the levels of concordance of detection of the tests used between parasitological, serological, and molecular data.

Reviewer #2: The methods described in this study are clear and properly referenced. There is however one question that could be addressed by the authors and that is: why was Trypanosoma theileri not included in the study? While being considered mostly non-pathogenic, the presence f this infection can lead to to false positive results in a number of diagnostic approaches.

Indeed *T. theileri* can be occasionally encountered in epidemiological surveys. It can be an issue in parasitological diagnosis for untrained technicians, but is otherwise easily identifiable. And in our particular study cannot be confused with a *T. vivax*. We did not specifically look for this parasite as it is considered apathogenic, and is rather ubiquitous, including in Europe. It is established that *T. theileri* infection does not interfere with serological tests. To our knowledge it is not picked up by ITS1 primers. At any rate, all pan-tryp positive samples were later found positive to one or more pathogenic trypanosomes. We added in the material and Methods section the statement

“No specific primers were used to detect *T. theileri*, this ubiquitous parasite being considered largely non-pathogenic.”.

Minor comment: could the TRYP1 primers be added to the primer table?
These primers are in the table, at the last row.

Results</br></br>
-Does the analysis presented match the analysis plan?</br>
-Are the results clearly and completely presented?</br>
-Are the figures (Tables, Images) of sufficient quality for clarity?</br></br>

Reviewer #1: Results and Discussion:
1. Table 2 presents a summary of the sampled animals per region, species and sex and n=1898 animals. Table 3 includes the various animal species that were sampled, classified according to their physical condition and n=1813.
R. Why are the numbers of total samples and animal species in Tables 2 and 3 different?

Some data related to age and/or physical conditions were not collected in the field, sometimes by mistake (for one cow), but in most cases because it concerns small ruminants or pigs, whose age is usually not known by the farmer, not being considered valuable animals, and physical condition is more difficult to estimate than in cattle. A comment was made below table 3:

“*this kind of data were not collected in the field for 85 animals, hence the difference in number with Table 2”.

In the following tables, which present summaries of BCT, PCR and ELISA results in the three study regions (Table 4) and the same results except for the classification by trypanosoma species (Table 5),
n= 1898.
R: Aside from the discrimination by trypanosome species, could the authors present the data about possible trypanosome co-infections based on their molecular diagnosis?

In table 4, we altered slightly the serological prevalence due to a small mistake in numbers. Afferent confidence intervals were also altered.

The table 6 of the original manuscript is now table 5, **PCR results regarding trypanosome species according to host**. We added the following statement for clarification:

“*Note that among the 16 (pigs) infected by *T.b* are the same 16 infected by *T.v* “

We replaced table 5 by table 6, **PCR results regarding trypanosome species according to regions,** to show as requested co-infections based on molecular

diagnostic. We removed co-infections based on serodiagnostics, this technique not being species-specific.

We created a Table 7, **ELISA results regarding trypanosome species according to regions,** to show serological results that were before combined with molecular results, as this technique does not allow to diagnose multiple infections as stated above.

This seems relevant, since the authors state in the Discussion (lines 430-432): “In our study, animals infected with one species of trypanosome had lower mean hematocrit than those diagnosed as negative, and animal with multiple infections even lower. See also reviewer’s next comment about seropositive animals for at least two species of trypanosomes.

We added a Table 8, PCV versus trypanosome species in cattle, to elaborate on this comment, and added a statistical analysis in the text.

“The average PCV of infected cattle was lower than that of uninfected cattle (χ^2= 60.5, df= 32, P= $1.7.10^{-3}$) (Table 8). That anemia is positively correlated with trypanosome infection is corroborated by the GLM analysis of molecular prevalence (Z= -2.06, P= 0.039). The lowest average PCV levels were observed for *T. brucei* infection (but P>0.05). Cattle with mixed infections appear with a lower anemia than those infected with a single species but this observation fails to pass statistical analysis (χ^2= 26.3, df= 29, P= 0.6) (Table 8).”

2. Lines 314-317: Concerning the seroprevalence, the authors indicate:
“Overall, of the 934 positive cases, 527 were T. vivax infections, while 59 were T. brucei infections, 26 were T. congolense and 323 (34.6%) cases were positive for at least two species of trypanosomes (Table 5).
R. The values (total and %) on Table 5 (%) do not match those of the above mentioned text.

As stated above, we removed references to co-infection based on serology. After consultation with specialists, if the OIE-ELISAs allow to determine species by comparison of relative positivity against the three species with a 80% confidence, it is not possible to assess multiple infections.

833 (43.9%) T. vivax, 325 (17.1%) T. brucei and 172 (9,1%) T. congolense.
In addition, there is no data in the table for co-infection with two trypanosome species.

These figures are caduc as “multiple infections” through serology cannot be considered in the study.

Reviewer #2: While the paper is very clearly written, the result section is very cryptic and most true scientific results are not shown. Hence, the reader is forced to 'accept' the conclusion of the authors, without being given a chance to appreciate the scientific data. Tis is OK for a review, but not really acceptable for scientific 'data'

publication.

We do agree with the referee that the data provided were inadequate. This was corrected and all available numerical data were now submitted.

Question 1: ELISA results in trypanosome research can be 'tricky' to interpret. The OD% explanation in the MM section makes one wonder what the real issue is the readout. This could be anywhere between 0.1 and 4.0 and while a % value will mask any inconveniences, it also makes the data very susceptible to individual interpretations. This reviewer understands that 10 plates of positive result is 'a lot' but the true results should be added as supplementary data. In particular for the 232 double PCR+ results. The reader should be given the chance to see 'what that means'.

This statement about ELISA results is totally legitimate. We have now provided not only the RPP (relative percent positivity), but also the ELISA readings of each plate to ponder. We used in this study the standard OIE (now WOAH) protocols used at CIRDES and CIRAD, as well as their reference sera. Both institutions are reference centres for the diagnosis of African Trypanosomoses.

The same holds true for the PCR data itself. While this reviewer understands that this is a lot of visual data, it is hard to accept that nothing is shown. For example: a reader could be interested in seeing how a PCR+/ELISA+ sample is scored n gel, versus a PCR+/ELISA- result. This data is important is the work is to be taken as a reference for others, and future studies.

As stated at the beginning of this document in a comment to the editor, we intended to label and submit for the reviewers' perusal all the 500+ gels used for the PCR, but it soon became obvious that this was a very daunting task. Instead, we provide as supplementary material some typical gels for each primer set.

A final questions: the results described in Lines 335- (the TL positive PCR positive T. brucei sample in pig and the T. b. gambiense positive sample in cattle: why are such important results mentioned without showing any true result. The authors must have the visual data in hand, so there is no reason not to show them (on not showing them, without mentioning 'data not shown' is truly not acceptable).

We are not sure what the reviewer implies as we stated that none of the samples were positive to *T.b. gambiense* primers by PCR, but to *T. brucei sensu lato* ones.

"The unique TL-positive pig was also PCR-positive for *T. brucei s.l.* on DNA extracted from both blood and biopsy. None of the TL-positive cattle were PCR-positive for *T. brucei gambiense*."

Conclusions</br></br>
-Are the conclusions supported by the data presented?</br>
-Are the limitations of analysis clearly described?</br>
-Do the authors discuss how these data can be helpful to advance our understanding

of the topic under study?</br>
-Is public health relevance addressed?</br></br>

Reviewer #1: Yes, the conclusions are supported by the data presented. The authors clearly state the limitations of the various tests performed, as well as the impact of other variables that may affect the trypanosome prevalence, such as the dry season, during which the sampling was carried out, the bias in the sampling towards animals in poor physical condition.
Yes, the authors addressed the importance of their study to better understand the dynamics of the epidemiology of AAT in Burkina Faso.

Reviewer #2: There are no major issues with the conclusions, other than that they cannot be challenged by the reader (or reviewer) because none of the actual data is shown. In fact, technically most of the 'result' section should be moved to the 'conclusion' section...leaving the actual scientific result/data section virtually empty.

Editorial and Data Presentation Modifications?</br>
Use this section for editorial suggestions as well as relatively minor modifications of existing data that would enhance clarity. If the only modifications needed are minor and/or editorial, you may wish to recommend “Minor Revision” or “Accept”.

Reviewer #1: Abstract:
Lines 31-32. Misspelling: It was therefore important to investigate the extend extent of the infection..
Corrected

Author abstract:
Line 59: The low occurrence of tsetse fly
R: Tsetse flies?
Corrected

Introduction:
Lines 81-82: The impact of trypanosomes on livestock productivity negatively affects millions of people in rural communities who depend on animal for their livelihood
R. Do the authors mean “animal products”?
We meant “animals”. Corrected

Lines 83-84: “adding a public health component to an already pregnant economic issue”
R. Suggestion:” adding a public health component to an already complicated economic issue”
Corrected

Lines 88-90: The potential benefits of better control of AAT on the continent, in terms of meat and milk productivity alone, are estimated to US$700 million per year (2).
R: are estimated up to US$700 million per year
Corrected

Lines 448-449 and 450-452:
The concordance of the results obtained with BCT and PCR confirms the specificity of these tests for the diagnosis of TAA.
The difference in specificity between this serological diagnosis and the others is high and its significance could be questioned for the diagnosis of active TAA infections.
R: Please, clarify the meaning of TAA, do the authors mean AAT?
Sorry, the (francophone) authors used the French acronym by mistake.

Reviewer #2: Data presentation?
See above...virtually no data is shown. Mostly conclusions are presented.

Summary and General Comments</br></br>
Use this section to provide overall comments, discuss strengths/weaknesses of the study, novelty, significance, general execution and scholarship. You may also include additional comments for the author, including concerns about dual publication, research ethics, or publication ethics. If requesting major revision, please articulate the new experiments that are needed.

Reviewer #1: The study provides a very good assessment of the epidemiology of AAT in Burkina Faso. The authors show excellent command of the strengths, weaknesses and limitations of the various diagnostic tools that were used. The combination and comparison of the various results as well as the statistical analyses performed to compare the data from three different regions, and various species of domestic animals are especially noteworthy and merit publication.
The manuscript requires minor revisions, as indicated above.

Reviewer #2: This is a paper with good potential. All this reviewer is asking for is to show the visual (PCR) and numerical (ELISA) data that has been used to draw the presented conclusions.
Data were provided in this resubmission. The limitations concerning visual PCR data are explained above.
The following line was added at the beginning of the result section.

“All numerical data used in this study can be accessed on
https://doi.org/10.18167/DVN1/OBRN6E”

PLOS authors have the option to publish the peer review history of their article (what does this mean?). If published, this will include your full peer review and any attached files.

If you choose “no”, your identity will remain anonymous but your review may still be made public.

Do you want your identity to be public for this peer review? For information

about this choice, including consent withdrawal, please see our Privacy Policy.

Reviewer #1: No

Reviewer #2: No

Figure Files:
While revising your submission, please upload your figure files to the Preflight Analysis and Conversion Engine (PACE) digital diagnostic tool, https://pacev2.apexcovantage.com. PACE helps ensure that figures meet PLOS requirements. To use PACE, you must first register as a user. Then, login and navigate to the UPLOAD tab, where you will find detailed instructions on how to use the tool. If you encounter any issues or have any questions when using PACE, please email us at figures@plos.org.

The figures were uploaded to PACE, and the file generated by the system was submitted with the manuscript, alongside the original figures.

Data Requirements:
Please note that, as a condition of publication, PLOS' data policy requires that you make available all data used to draw the conclusions outlined in your manuscript. Data must be deposited in an appropriate repository, included within the body of the manuscript, or uploaded as supporting information. This includes all numerical values that were used to generate graphs, histograms etc.. For an example see here: http://www.plosbiology.org/article/info%3Adoi%2F10.1371%2Fjournal.pbio.1001908#s5.

All data were submitted to https://dataverse.cirad.fr/dataset.xhtml?persistentId=doi:10.18167/DVN1/OBRN6E

Reproducibility:
To enhance the reproducibility of your results, we recommend that you deposit your laboratory protocols in protocols.io, where a protocol can be assigned its own identifier (DOI) such that it can be cited independently in the future. Additionally, PLOS ONE offers an option to publish peer-reviewed clinical study protocols. Read more information on sharing protocols at https://plos.org/protocols?utm_medium=editorial-email&utm_source=authorletters&utm_campaign=protocols

All protocols used in this study come from the COMPENDIUM OF STANDARD DIAGNOSTIC PROTOCOLS FOR ANIMAL TRYPANOSOMOSES OF AFRICAN ORIGIN, Montpellier, October 2017. Marc Desquesnes et al. Available on the OIE / WOAH website following the link https://www.woah.org/nttat/Attached%20files/A16-REC-COMPENDIUM_PROTOCOLES_TRYPANO-En.pdf.

In compliance with data protection regulations, you may request that we remove your personal registration details at any time. (Use the following URL: https://www.editorialmanager.com/pntd/login.asp?a=r). Please contact the publication office if you have any questions.
