# Supplementary material for: Molecular epidemiology of Animal African Trypanosomosis in southwest Burkina Faso
Source: PLoS Negl Trop Dis. 2022 Aug 22;16(8):e0010106. doi: 10.1371/journal.pntd.0010106 (PMC9436040; doi:10.1371/journal.pntd.0010106)
Supplement: Attachment — Submitted filename: renamed_0261b.pdf [file pntd.0010106.s002.pdf]

Dear Members of the editorial board,

Please find here a further revised version of the manuscript "Molecular epidemiology of Animal African Trypanosomosis in southwest Burkina Faso" for consideration at PLOS Neglected Tropical Diseases. We have modified the manuscript according to the review recommendations.

You will find below a detailed list of our responses to all review comments, and a description of the changes we have made in the manuscript.

Hoping that this version will meet your approval,

Yours sincerely

Alain Boulangé

Dr. Alain Boulangé, Project Coordinator
CIRAD, UMR 17 Intertryp
Institut Pierre Richet - INSP
Bouaké, Côte d'Ivoire
Cell/WhatsApp: +225 07 78 19 71 69
COMBAT COntrolling and progressively Minimizing the Burden of Animal Trypanosomosis
www.combat-project.eu
https://doi.org/10.12688/openreseurope.14759.1

****

Lines 278-280: "The overall molecular prevalence assessed by PCR was 23.1% with a total of 438 positive samples. The Hauts-Bassins region showed the highest prevalence with 60% followed by Sud-Ouest 12% and Cascades 10.6% with 276, 80 and 82 positive animals respectively 280 (Table 4).
R: The authors must revise and correct either the text or the data in Table 4. If the values presented in Table 4 are correct, the text should read: "The Sud-Ouest region showed the highest prevalence with 60%, followed by Hauts-Bassins with 12% and Cascades with 10.6% with 276, 80 and 82 positive animals respectively (Table 4).

This is correct, we inverted by mistake Sud-Ouest and Hauts-Bassins in the text. The table has the correct values. This was changed in the text.

Line 360. Column heading for Table 8. PCV versus trypanosome species in cattle

R: The column heading "Nbre d'animaux" must be translated to English.

Apologies, that was done.

Lines 368-370: "In details, the Hauts-Bassins region presented the highest AD with 2.32 flies caught per trap per day followed by Sud-Ouest and Cascades regions with 0.42 and 1.23 respectively".

R: The statement should be corrected to present the regions in decreasing order of AD:

"In details, the Hauts-Bassins region presented the highest AD with 2.32 flies caught per trap per day, followed by Cascades and Sud-Ouest regions with 1.23 and 0.42, respectively".

Corrected in the text.

Lines 80-81: "In addition, the same tsetse flies also transmit the agents of Human African Trypanosomosis (HAT), also known as sleeping sickness (5, 6)."

R: Replace the term "Trypanosomosis" for the correct one for human disease: "Trypanosomiasis".
"In addition, the same tsetse flies also transmit the agents of Human African Trypanosomiasis (HAT), also known as sleeping sickness (5, 6)"

We agree to the request to use this form in -iasis to refer to HAT, and the change was made in the text.

Line 474: A reference of a very recently published paper was added (Boulangé A, Lejon V, Berthier D, et al. The COMBAT project: controlling and progressively minimizing the burden of vector-borne animal trypanosomosis in Africa. Open Res Europe 2022, 2:67.), that presents an on-going research project in which frame fits the type of methodology and results presented in our paper fit perfectly. Although of wider reach, we cited it in reference to the Atlas, whose update will be in the context of the project. We want to state that the purpose of this addition is not to put an auto-citation, but rather because we think the readers of the present article would be interested to know that a continent-wide initiative to tackle animal trypanosomosis exists.
